# Knowledge, attitude and preventive practices of COVID-19 among deaf persons in the Greater Accra region of Ghana

**DOI:** 10.1186/s12889-023-15818-1

**Published:** 2023-05-26

**Authors:** Reginald Arthur-Mensah Jnr, Jacob Nartey Quao, Louisa Yeboah, Zanu Dassah, Abigail Agartha Kyei

**Affiliations:** 1 Department of Nursing and Midwifery, Faculty of Health and Allied Sciences, Pentecost University, Accra, Ghana; 2 Ayawaso North Municipal Health Directorate, Ghana Health Service, Accra, Ghana; 3 Sign Language Unit, Maamobi General Hospital, Accra, Ghana; 4 Faculty of Education, Pentecost University, Accra, Ghana; 5 Out Patient Department, Ga North Municipal Hospital, Accra, Ghana; 6 Human Resource Division, Ghana Health Service, Accra, Ghana

**Keywords:** Attitude, Ayawaso North Municipality, COVID-19, Deaf persons, Ghana, Knowledge, Practice

## Abstract

**Background:**

Since the emergence of the COVID-19 pandemic, studies continue to investigate the KAP of COVID-19 among diverse groups. We examined the KAP of COVID-19 among deaf persons living in the Ayawaso North Municipality in Accra.

**Methods:**

A descriptive cross-sectional design was used for this study. Our sample comprised deaf persons registered with the Municipal Directorate. In all, 144 deaf persons were interviewed using an adapted KAP COVID-19 questionnaire.

**Results:**

Regarding knowledge, majority of the deaf persons (> 50%) were not in the know of 8 out of 12 items of the knowledge subscale. For attitude, deaf persons (> 50%) showed optimistic attitude in all 6 items of the attitude subscale. Deaf persons “always” practised 5 items and “sometimes” practised 4 items in the preventive practices to COVID-19. A positive moderate and significant correlation existed between the subscales. Regression analysis showed that, a one-unit increase in knowledge will result in a 1.033-unit increase in preventive practices while a one-unit increase in knowledge will result in a 0.587-unit increase in attitude.

**Conclusions:**

Campaigns about COVID-19 should emphasize the teaching of the science of the virus and the disease and not just the preventive practices, paying special attention to deaf persons.

## Background

The novel coronavirus disease 2019 (COVID-19) is the latest in the list of pandemics that is reforming every level of human survival. COVID-19 is an infectious disease caused by the severe acute respiratory syndrome coronavirus 2 (SARS-CoV-2) virus [1]. The WHO declared the COVID-19 disease outbreak a public health emergency of international concern on 30th January 2020, and a pandemic on 11th March 2020 [2].

SARS CoV-2 belongs to the subfamily *Coronavirinae* in the family of Coronaviridae. The genome of CoVs is a single-stranded positive-sense ribose nucleic acid (+ ssRNA) [3]. Generally, coronaviruses cause respiratory, digestive, and nervous system diseases in humans and many other animals [4].

The virus spreads through small liquid particles from an infected person’s mouth or nose when they cough, sneeze, speak, sing or breathe out. These liquid particles range from large respiratory droplets to small aerosols [5].

Anyone can get infected with the virus and become ill at any time. However, most people infected with the virus will experience mild to moderate respiratory illness and recover without requiring special treatment. Others will become seriously ill and require medical attention. Older people and those with underlying medical conditions like cardiovascular disease, diabetes, chronic respiratory disease, hypertension and cancer are more likely to develop serious illness [6–8].

Symptoms of the disease have been classified into three levels according to the severity of the disease; the less common symptoms, the most common symptoms and serious symptoms. The less common symptoms include experiencing sore throat, headaches, aches and pains, diarrhoea, noticing a rash on skin, or discoloration of fingers or toes red or irritated eyes. The most common symptoms include experiencing fever, tiredness, loss of taste or smell and having a dry cough. The serious symptoms include difficulty breathing or shortness of breath, loss of speech or mobility, or confusion and chest pains [9].

On average it takes 5–6 days from when someone is infected with the virus for symptoms to show up, however it can take up to 14 days for symptoms to show up. Important complications due to the infection include acute respiratory distress syndrome, sepsis, digestive stress, liver injury, hyperinflammatory response, multiorgan failure, thromboembolism and vascular damage [10, 11].

The following current precautionary measures are recommended according to public health standards; wearing face protection in high risk environments e.g., properly fitting surgical mask, practicing physical distancing (at least 1 m apart) from others, practicing good respiratory hygiene by covering the mouth and nose with a cloth when sneezing or coughing or sneezing or coughing in your flexed elbow in the absence of a cloth, choosing open, well-ventilated spaces over closed ones, washing the hands regularly with soap under running water for at least 30 s and cleaning the hands intermittently with an alcohol-based hand sanitizer. The others include staying home or indoors if one is experiencing any of the symptoms and getting vaccinated [12–14].

Global observations have shown that the success or failure in preventing and controlling the spread of the disease largely relies on human behaviours [12]. This is dependent on the level of knowledge and/or perception of the disease, the attitudes adopted by persons due to the level of knowledge, information and/or perception of the disease and the decision to adhere to the preventive practices (KAP) of the disease. These can be examined via the KAP framework [15]. Knowledge is factual information that a person identifies with. It is also a way of conceiving something borne from an individual’s perceiving. Attitude involve beliefs that predisposes individuals to act in certain ways. They are intermediary between the response to any phenomenon. They reveal the variable stands for an individual submitted to any stimulus which can be directly or indirectly observed and assessed. Practices or behaviours are the observable actions of an individual in response to a stimulus [15, 16]. Generally, the framework provides information about what is already known, perceived, believed and done about a phenomenon of interest among a target population. The findings then reveal misunderstandings, misconceptions and helps to locate aspects where information and education efforts remains to be maximized, and misbehaviors that signify hindrances to the phenomenon of interest [15]. The framework hypothesizes that, knowledge that is considered beneficial translates into optimistic attitude and the practice of the knowledge [16].

Since the emergence of the pandemic, studies have been conducted to investigate the KAP on the novel COVID-19 among diverse groups including community dwelling adults, parents, health care workers, students etc. [17–22]. These studies have allowed us to identify groups that have adequate or deficient knowledge of the disease, groups that have developed positive or negative attitudes to the disease and groups that are adhering to or not to the preventive practices of the disease. By and large, to facilitate effective management of the pandemic globally, an all-inclusive population awareness of the KAP of the disease are fundamentally important. We therefore set forth to contribute to the scientific literature on the scope of COVID-19 by examining the KAP of COVID-19 among deaf persons living in the Ayawaso North Municipality of the Greater Accra region of Ghana.

## Methods

### Research design

The design of this study was a descriptive cross-sectional design utilizing a quantitative approach. The purpose of descriptive studies is to provide information about a naturally occurring phenomenon in any sample or population of interest [23]. Cross-sectional studies involve a current description of any phenomenon in a sample or population of interest [24]. Though they might not establish causal relationships between variables, descriptive cross-sectional studies show associations between variables of a study and the extent of a phenomenon in the sample or population of interest [25]. Accordingly, this study provided information about the KAP of COVID-19 among a sample of deaf persons living in the Ayawaso North Municipality of the Greater Accra region of Ghana.

### Study area

The Ayawaso North Municipality (Latitude 5.591818; Longitude − 0.199017) is one of the 29 Metropolitan, Municipal and District Assemblies (MMDAs) in the Greater Accra region of Ghana. It is bounded to the North by Ayawaso Central (Pig Farm), to the South by Ayawaso East (Nima), to the East by Ayawaso Central (Accra Newtwon) and the West by Ayawaso West (Dzorwulu). The vegetation of the area is mainly savanna grassland. The housing structures comprise detached, semi-detached and compound houses. There are two main rainfall patterns, the major one from May to July and the minor one from September to October. The dry season is experienced from November to February. Occupation of the residents are petty trading for the women and artisanship for the men. Some major public and private institutions are also situated in the Municipality. The Maamobi General Hospital was the location for COVID-19 vaccination for residents in the municipality.

The deaf persons live among the hearing population in the Municipality. Some live alone or with their friends and relatives and some have families. The hearing population who know about them are mindful to interact with them according to their deaf culture.

### Study population

Deaf persons registered with the Municipal Directorate and living in the Ayawaso North Municipality were sampled for the study. They included both male and female deaf persons above the age of ten years who could communicate the American/Ghanaian Sign Language (A/GSL).

### Sample size and sampling procedure

The census method of sampling was used to include all deaf persons registered with the Municipal Directorate. In all, 144 deaf persons were sampled.

### Data collection instrument

The KAP COVID-19 instrument developed by Park [26] was adapted for this study. Our KAP COVID-19 questionnaire consisted of 12 question items on knowledge, 6 question items on attitude and 9 question items on adherence to preventive practices of COVID-19 (see Results section). Responses were measured on a numeric rating scale. Question items on knowledge were rated as I do not know = 0 and Yes = 1. Question items on attitudes were rated as No = 0, I am not sure = 1 and Yes = 2. Question items on adherence to preventive practices of COVID-19 were rated as Never = 0, Sometimes = 1, Often = 2 and Always = 3.

### Data collection procedure

Data collection was scheduled during the second round of the National COVID-19 mass vaccination at the Municipality. These second round of vaccinations was to help achieve herd immunity against the virus in the country. A cubicle for our data collection was setup alongside the National COVID-19 mass vaccination team setup during the second round of vaccination. As deaf persons came to the center to be registered for the vaccination, we collected our data before they received the jab. In the cubicle, they were warmly welcomed, given a seat and served with water. The purpose of the study was then thoroughly explained to deaf persons. Afterwards, they were given opportunity to ask questions and seek clarification on any issue relating to the study. Those who agreed to take part in the study, signed or thumb printed the consent forms before data was collected. Some questionnaires were self-administered whilst others were completed with the help of the researchers.

Notably, data was collected by researchers who were familiar with the deaf culture, thus, all values peculiar to the deaf culture were observed. All communication was done using the A/GSL. Additionally, COVID-19 protocols were observed between the researchers and the deaf persons during the period of data collection. After data collection, deaf persons proceeded to take their second jab of COVID-19 vaccine. Data was collected from April 19th to 30th, 2022.

### Data analysis

Data analysis was performed using IBM SPSS software version 23.0 (SPSS Inc, Chicago, IL, USA). Firstly, frequencies and percentages were used to present the demographic details of our sample. Secondly, frequencies and percentages were used to present the responses to the knowledge and attitude subscales. Average scores using modal values were used to present the results of the adherence to the preventive practices of COVID-19 subscale. Correlation analysis was performed to determine the direction and strength of the KAP subscales in relation to the responses of our sample. Lastly, regression analysis was performed to determine the effect of the independent variable, knowledge, on the dependent variables, attitude and preventive practices to COVID-19. Correlation and regression analysis were considered significant at$P\le 0.01.$

## Results

### Demographic details of deaf persons

A total of 144 deaf persons were sampled. They comprised 68/144 (47.2%) males. Their mean age was 36 years (± 12.84). The range of their ages was from 12 years to 65 years. Regarding their educational attainment, the majority, 48/144 (33.3%) attained up to Junior High School level of education. Around 19.4% had no formal education. About a quarter of the deaf persons, 26/144 (18.1%) had attained up to the Basic level and the Senior High School level of education and 16/144 (11.1%) had attained up to the tertiary level of education. The bulk, 64/144 (44.4%) of the deaf persons were unemployed, 40/144 (27.8%) were doing their own businesses, 24/144 (16.7%) were working at various private sector businesses and 16/144 (11.1%) were Government workers. About 80.6% of deaf persons were unmarried (Table 1).

**Table 1:** Demographic details of Deaf persons (n = 144)

Demographic details	*f* (%)
Gender	
Males	68 (47.2)
Females	76 (52.8)
**Educational level**	
No formal education	28 (19.4)
Primary	26 (18.1)
Junior High School	48 (33.3)
Senior High School	26 (18.1)
Tertiary	16 (11.1)
**Occupation**	
Unemployed	64 (44.4)
Government worker	16 (11.1)
Private business employee	24 (16.7)
Doing own business	40 (27.8)
**Marital status**	
Married	28 (19.4)
Unmarried	116 (80.6)

### Level of knowledge of COVID-19 among deaf persons

Results showed that majority of the deaf persons were not knowledgeable in 8 out of 12 questions items in the knowledge subscale. This represented a lack of knowledge of 67% of the items of the knowledge subscale (Table 2).

**Table 2:** Level of knowledge of COVID-19 among Deaf persons (n = 144)

Question items	Response option	*f* (%)
1. COVID-19 is a disease caused by a virus	**I do not know**	**124 (86.1)**
	Yes	20 (13.9)
2. The virus is an RNA virus	**I do not know**	**138 (95.8)**
	Yes	6 (4.2)
3. The virus is called SARS COV-2	**I do not know**	**140 (97.2)**
	Yes	4 (2.8)
4. On average, it takes 5–14 days when someone is infected with the virus for symptoms to show up	**I do not know**	**130 (90.3)**
	Yes	14 (9.7)
5. COVID-19 is currently being diagnosed using antibodies, proteins and gene detection methods	**I do not know**	**140 (97.2)**
	Yes	4 (2.8)
6. Current treatment for COVID-19 is symptomatic treatment	**I do not know**	**90 (62.5)**
	Yes	54 (37.5)
7. To this date, there is no antiviral agent specific to the virus.	**I do not know**	**122 (84.7)**
	Yes	22 (15.3)
8. The virus keeps mutating	**I do not know**	**94 (65.3)**
	Yes	50 (34.7)
9. COVID-19 is spread through droplet transmission	I do not know	46 (31.9)
	Yes	98 (68.1)
10. Anyone can get infected with the virus	I do not know	22 (15.3)
	Yes	122 (84.7)
11. The main clinical symptoms of COVID-19 include fever, dry cough, tiredness, loss of taste and smell	I do not know	24 (16.7)
	Yes	120 (83.3)
12. Persons with underlying health conditions might suffer serious illness of the disease.	I do not know	60 (41.7)
	Yes	84 (58.3)

The bold texts within the table were employed to draw attention to those aspects of the results.

### Attitude of deaf persons towards COVID-19

Results showed that deaf persons had good attitude towards COVID-19. Majority of Deaf persons showed optimistic attitude in all 6 items of the attitude subscale. As a point worthy of note, all deaf persons said “yes” to getting vaccinated against COVID-19 on the day of data collection and any other time and were ready to encourage their family and friends to get vaccinated against COVID-19 (Table 3).

**Table 3:** Attitude of Deaf persons towards COVID-19 (n = 144)

Question items	Response option	*f* (%)
1. I believe that COVID-19 can be prevented if I adhere to the preventive guidelines	No	4 (2.8)
	I am not sure	16 (11.1)
	**Yes**	**124 (86.1)**
2. I encourage others to adhere to the preventive guidelines	No	2 (1.4)
	I am not sure	22 (15.3)
	**Yes**	**120 (83.3)**
3. I will go and test for COVID-19 if need be	No	2 (1.4)
	I am not sure	46 (31.9)
	**Yes**	**96 (66.7)**
4. I will encourage my family and friends to test for COVID-19	No	2 (1.4)
	I am not sure	50 (34.7)
	**Yes**	**92 (63.9)**
5. I will go and get vaccinated against COVID-19 today and any other time	No	0
	I am not sure	0
	**Yes**	**144 (100)**
6. I will encourage my family and friends to get vaccinated against COVID-19	No	0
	I am not sure	0
	**Yes**	**144 (100)**

The bold texts within the table were employed to draw attention to those aspects of the results.

### Adherence to preventive practices of COVID-19 among deaf persons

Average score using modal values were used to represent the aggregate responses of our sample. Results showed that deaf persons “always” practised 5 items and “sometimes” practised 4 items in the adherence to preventive practices subscale. This represented a strict adherence to the preventive practices of COVID-19 of 56% of the items of the adherence to COVID-19 preventive practices subscale (Table 4).

**Table 4:** Adherence to preventive practices of COVID-19 among Deaf persons

Question items	Modal score
1. I wear face protection at all times	3.00
2. I avoid physical contact (e.g., handshakes, hugs, etc.) with others as much as possible	3.00
3. I choose open, well-ventilated spaces over closed ones	3.00
4. I wash my hands regularly with soap under running water for at least 30 s	3.00
5. I clean my hands intermittently with an alcohol-based hand sanitizer	3.00
6. I practice physical distancing (at least 1 m apart) from others, even if they do not appear sick as much as possible	1.00
7. I practicing good respiratory hygiene by covering my mouth and nose with a cloth when sneezing or coughing or I sneeze or cough in my flexed elbow in the absence of a cloth	1.00
8. I avoid touching my eyes, nose and mouth as much as possible	1.00
9. I stay home or indoors if I am experiencing any of the symptoms and any other symptoms, I am not sure of	1.00

Correlation analysis was performed to determine the direction and strength of the KAP subscales in relation to the responses of our sample. Results showed a positive moderate and significant correlation between the subscales (Table 5). Regression analysis was then performed to determine the effect of the independent variable, knowledge, on the dependent variables, attitude and preventive practices to COVID-19. The first model showed that a one-unit increase in knowledge will result in a 1.033-unit increase in preventive practices to COVID-19. The second model also showed that a one-unit increase in knowledge will result in a 0.587-unit increase in attitude (Table 6). This depicts that if deaf persons had knowledge on most of the questions items under the knowledge subscale, it would have resulted in much more unit increases in their attitude and preventive practices scores to COVID-19.

**Table 5:** Correlation analysis to determine the direction and strength of the KAP subscales

		Preventive practices	Attitude	Knowledge
Preventive practices	Pearson Correlation	1	0.440^**^	0.439^**^
	Sig. (2-tailed)		0.000	0.000
Attitude	Pearson Correlation	0.440^**^	1	0.547^**^
	Sig. (2-tailed)	0.000		0.000
Knowledge	Pearson Correlation	0.439^**^	0.547^**^	1
	Sig. (2-tailed)	0.000	0.000	

** indicates correlation is significant at P ≤ 0.01.

**Table 6:** Regression analysis to determine the effect of the independent variable, knowledge, on the dependent variables, attitude and preventive practices to COVID-19.

Model		Unstandardized Coefficients		Standardized Coefficients	t	Sig.
		B	Std. Error	Beta		
	Dependent Variable: Preventive practices					
1	(Constant)	14.057	0.826		17.024	0.000
	Knowledge	**1.033**	0.177	0.439	5.830	0.000
	Dependent Variable: Attitude					
1	(Constant)	1.854	0.352		5.268	0.000
	Knowledge	**0.587**	0.076	0.547	7.777	0.000

## Discussion

Having observed with ardent interest in the scientific literature on the scope of the COVID-19 disease since its inception, specifically on the KAP of the disease, we noticed few reports on the KAP of COVID-19 among deaf persons globally. We therefore set forth to contribute to the literature on the scope of COVID-19 by examining the KAP of COVID-19 among deaf persons in the Ayawaso North Municipality of the Greater Accra region of Ghana.

Good attitude and adherence to the preventive practices of COVID-19 was observed among the deaf persons. However, a recent review on communication challenges among individuals with hearing impairments/deafness during the COVID-19 pandemic, mainly with evidence from the US and UK reported poor knowledge regarding the preventive practices to COVID-19 among deaf persons [27]. The good attitude and adherence to the preventive practices of COVID-19 observed in our study, reflects the mass public health prevention campaigns to COVID-19 by various stakeholders including the Ministry of Health (MOH) Ghana, Ghana Health Service (GHS), Ministry of Information (MOI), media houses and some individuals. All campaigns about the disease including the periodic ‘presidential updates on measures taken in the fight against the pandemic in Ghana’, largely focused on the preventive practices against the disease. Besides, the GHS through it Municipal Health Directorates (MHD) routinely organized targeted public health prevention campaigns against COVID-19 for deaf persons registered with the municipalities. In these campaigns, deaf persons were constantly updated on aseptic ways to handle, wear and dispose of their facemasks, the right steps to wash their hands, the right steps and moments to use hand sanitizers, how to practice physical distancing, how to practice good respiratory hygiene and to get vaccinated when the vaccines arrive in the country.

As is the case, targeted special public health education and service about the COVID-19 vaccination was done by GHS through its MHDs for all deaf persons registered with the municipalities when the second dose of COVID-19 vaccines were received in the country and it was time to vaccinate the populace. It was during one of such provisions for deaf persons, i.e., the nationwide second dose of COVID-19 vaccination for deaf persons, that this study was conducted. Additionally, very early in the pandemic, during televised live briefings about the situation of the pandemic in the country by the President or the Minister for Information or the Minister for Health or the Director of the GHS, sign language interpreters signed during these broadcast. These broadcasts also never ended without reiterating the adherence to the preventive practices against the disease [28]. Other COVID-19 outreaches by various organizations, also had sign language interpreters as part of the team.

Regarding the knowledge of the science of COVID-19, the MHD were shown the findings of this study. They agreed to it, realizing their primal goal has been on teaching deaf persons on how to protect themselves from the disease rather than the science of the disease. Specifically, deficient knowledge was found on the causative agent of the disease, the nature and dynamics of the virus, the name of the virus, the incubation period of the virus and the detection and diagnostic means of the virus. This is disturbing because these items represent the basic information about the disease. One of the major concerns about the pandemic was ensuring access to right information about the virus and the disease to all persons [12]. Concerning deaf persons, many communication gaps sort to be bridged by various advocacy institutions for deaf persons working in partnership in this period. One of such interventions was the development of new vocabulary for COVID-19 terminology for deaf persons [29]. This helped the deaf persons and sign language interpreters engaged in various formal and informal interpretation of COVID-19 related information resources. Yet still, this study shows that gaps still persist. A relational barrier may be seen here. On the part of the sign language interpreters, it may be likely that interpreters were not keeping themselves abreast with the international sign language for COVID-19 or interpreters were unable to break down the scientific terminologies e.g., SARS COV-2, RNA, droplet infection, underlying health conditions, antibodies, proteins and gene detection methods, mutation, herd immunity etc. associated with the disease to the deaf persons. On the part of deaf persons too, it is probable that deaf persons did not understand or were unfamiliar with the scientific terminologies associated with the disease. In such situations, interpreters must use a scenario to describe what they are communicating or finger spell what they are describing to deaf persons. These are in harmony with [27] who identified improving the availability of sign language interpreters and teaching basics of sign language to health care professionals as mitigating strategies to the challenges deaf persons experienced in the pandemic. The low educational level recorded amongst deaf persons in our study could have influenced their appreciation of these scientific terms or rather simply, deaf persons were also not keeping themselves informed and updated on the science of the disease.

The knowledge of the disease, the attitudes adopted by persons due to the level of knowledge of the disease and the decision to adhere to the preventive practices (KAP) of the disease among all population groups is vital in the control and management of the disease in Ghana and globally.

### Limitations of the study

The current assessment was conducted among deaf persons in the Ayawaso North Municipality of the Greater Accra region. Other deaf persons in other Municipalities in the Greater Accra and in Ghana remain to be sampled. As such, the findings of this study should be interpreted in this scope. The design of the study limits the predictive strength of the variables studied among our sample. As well, causal relationship between variables cannot be established.

## Conclusions

The glaring challenges to social inclusion posed by the pandemic was examined in terms of the KAP of COVID-19 among deaf persons in Ghana. The results have shown the need for exhaustive and public health education about COVID-19 in light of the respective population involved. Educational campaigns about COVID-19 should emphasize the teaching of the science of the virus and the disease and not just the preventive practices, paying special attention to deaf persons. New concepts and vocabulary relating to health concepts especially COVID-19 should be published far and wide regularly to keep the deaf persons and sign language interpreters updated, to ensure effective communication. Health institutions should continually employ the services of qualified sign language interpreters to guarantee the incessant inclusion of deaf persons in health activities.

## Data Availability

The data that support the findings of this study are available from Municipal Health Directorate of the Ayawaso North Municipality but restrictions apply to the availability of these data due to our study sample, which were used under license for the current study, and so are not publicly available. Data are however available from the corresponding author upon reasonable request and with permission from the Municipal Health Directorate of the Ayawaso North Municipality.

## List of abbreviations

A/GSL: American/Ghanaian Sign Language
COVID-19: Coronavirus disease 2019
GHS: Ghana Health Service
KAP: Knowledge, Attitude, Practice
MoH: Ministry of Health
SARS CoV-2: Severe Acute Respiratory Syndrome Coronavirus 2
WHO: World Health Organization
